# Interoceptive sensibility differentiates the predictive pattern of emotional reactivity on depression

**DOI:** 10.3389/fpsyg.2023.1011584

**Published:** 2023-03-02

**Authors:** Yun-Hsin Huang, Yu-Ting Huang, Nai-Shing Yen

**Affiliations:** ^1^Department of Psychology, Fo Guang University, Yilan, Taiwan; ^2^Research Center for Mind, Brain, and Learning, National Chengchi University, Taipei, Taiwan; ^3^Department of Psychology, National Chengchi University, Taipei, Taiwan

**Keywords:** Emotional reactivity, Depression, Interoception, Interoceptive awareness, Interoceptive sensibility

## Abstract

The role of emotional reactivity in the psychopathology of depression has been studied widely but not comprehensively. Inconsistencies in existing literature indicate the presence of other factors may affect this dynamic. An individual’s method of processing their physiological sensations is a third variable because emotions are psychophysiological. This study identified the predictiveness of ease of activation, intensity, and duration of negative and positive emotions on depressive symptoms differentiated by interoceptive sensibility (IS). A total of 270 community participants filled-in questionnaires assessing their IS, habitual emotional reactivity, depressive severity, and response bias. A two-step clustering analysis identified the IS characteristics. Negative and positive reactivity models among each IS cluster were tested using bootstrapping regression, controlling for gender and response bias. IS can be clustered into “high IS,” “low IS,” and “worriers.” Both positive and negative reactivity’s predictiveness patterns on depression were different between IS clusters. Lower positive reactivity predicted depression among individuals with low IS (harder to activate positive emotions) and worriers (shorter duration of positive emotions) but not among individuals with high IS. Those with high IS also exhibited the highest positive reactivity. Ease of activating negative emotions predicted depression among high IS individuals, and a longer duration of negative emotions predicted depression among worriers. IS may affect the psychopathology of depression through subjective emotional reactivity. Thus, IS characteristics can be incorporated into intervention plans.

## 1. Introduction

The psychopathology of depression has been studied from the perspective of affective style. However, this approach is far from complete. Various studies have explored how reactions to positive and negative stimuli contribute to depressive disorders. Some studies have examined physiological reactivity to designed stimuli in the laboratory. Most of these revealed that blunted reactivity (e.g., heart rate and heart rate variability change, skin conductance change) to both positive and negative stimuli is associated with depressive problems (e.g., Peeters et al., 2003; Rottenberg et al., 2005; Bylsma et al., 2008; Schwerdtfeger and Rosenkaimer, 2011; Shinba, 2014; Liang et al., 2015; Hill et al., 2019; Kim et al., 2019). Rottenberg et al. (2005) proposed emotion context insensitivity (ECI), which suggests a general blunting of emotional reactivity in depression associated with disengagement and lack of motivation. Studies that used the experience sampling method (ESM) recorded daily events and reactions to events through participants’ self-reports. Inconsistent with the laboratory findings, these ESM studies sometimes found a decreased positive reactivity and increased negative reactivity (Myin-Germeys et al., 2003; Peeters et al., 2003; Bylsma et al., 2011), supporting cognitive theories, such as one which posits blunted positive and exaggerated negative reactivity due to negative schema (Beck, 1979; Gross and Jazaieri, 2014). However, both entailed immediately addressing short-term responses after a single stimulus and did not focus on the comprehensive dimensions of emotional reactivity.

Emotional reactivity should be investigated in a multifaceted manner. Davidson (1998) proposed several important features of affective style, including the threshold for reactivity, peak amplitude of response, rise time to peak, and recovery time. Various components may function differentially in psychopathology (Davidson, 2000; Hofmann et al., 2012). For example, Rottenberg et al. (2007) found a lower peak amplitude (i.e., less vagal withdrawal) and recovery difficulty under stress among individuals with depression compared to their controls. However, such differential investigations were mainly conducted in a laboratory and lacked data on positive reactivity. It is also difficult to further differentiate the threshold for reactivity from the peak amplitude of the response in existing literature. Investigating emotional reactivity using differentiated dimensions will provide a better understanding of the psychopathology of depression. By adapting Davidson’s affective style, Becerra et al. (2017) developed the Perth Emotional Reactivity Scale (PERS) to assess the emotional reactivity features in a habitual trait-like level. Becerra et al. (2017) suggested that individuals may differ in terms of how easily and quickly they can be emotionally activated (activation), how strong their emotional responses are (intensity), and how long their emotional responses last (duration). Investigating how depressive levels differ according to individual differences across these three dimensions will provide deeper insights into the role of emotional reactivity in the psychopathology of depression.

Other factors can also be considered to address the debate between ECI and cognitive theories. Individual differences in other components of the emotional process may also affect emotional reactivity functions. Hershenberg et al. (2017) found that both positive and negative emotional reactivity were reduced among depressive veterans with greater experiential avoidance but not among depressive veterans with lower experiential avoidance. Along with the tendency of avoiding one’s own experiences, the tendency of attending to one’s own experiences also might play a role in such a process. Thus, we proposed interoceptive sensibility (IS) as a potential factor that may differentiate the relationship between emotional reactivity and depression. This is because IS addresses individual differences in terms of attention, interpretation, and manipulation of one’s physiological sensations, and it is highly relevant to the emotional process.

The manner in which individuals mentally process their own physiological reactions is hypothesized as an important component of psychopathology (Khalsa et al., 2018). Interoception is defined as “sensing the physiological condition of the body, as well as the representation of the internal state within the context of ongoing activities, and is closely associated with motivated action to homeostatically regulate the internal state” (Herbert and Pollatos, 2012 p. 693). Peripheral theories suggest emotion is rooted in physiological responses, and the sensations and perceptions are organized as a whole, then cognitively translated into “feelings” (James, 1922; Friedman, 2010; Levenson, 2014). Thus, interoception, by its nature, is closely related to emotional processes. Supporting this idea, various operations of interoception have been found to be related to emotion regulation or regulation difficulty, alexithymia, anxiety problems, post-traumatic stress disorder, eating disorders and obesity, and depression (for a review, see Khalsa et al., 2018). Within this broad spectrum, IS was recently introduced to assess the purely subjective domain, that is, the dispositional characteristics of noticing, focusing on, appraisal, and action of interoceptive stimuli, which are highly involved with beliefs and attitudes (Mehling et al., 2012; Khalsa et al., 2018; Mehling et al., 2018; Pollatos and Herbert, 2018). IS has been found to be associated with emotion regulation or regulation difficulty (Mehling et al., 2012; Zamariola et al., 2019; Schuette et al., 2021), alexithymia (i.e., difficulty in recognizing emotions) (for a review, see Trevisan et al., 2019). Hence, IS may differentiate the relationship between emotional reactivity and depression through individual differences in the processing of physiological reactivity into subjective reactivity induced by emotional stimuli.

IS is often assessed using the Multidimensional Assessment of Interoceptive Awareness (MAIA). It was developed by integrating constructs assessed in previous body awareness scales (Mehling et al., 2009), thus covering the widest dimensions of IS, including attention, interpretation, and manipulation of bodily sensations (Mehling et al., 2012). It contains eight subscales: (1) noticing, i.e., awareness of uncomfortable, comfortable, and neutral body sensations; (2) not-distracting, i.e., the tendency to not ignore or distract oneself from sensations of pain or discomfort; (3) not-worrying, i.e., the tendency to not worry or experience emotional distress with sensations of pain or discomfort; (4) attention regulation, i.e., the ability to sustain and control attention to bodily sensations; (5) emotional awareness, i.e., awareness of the connection between body sensations and emotional states; (6) self-regulation, i.e., the ability to regulate distress by paying attention to bodily sensations; (7) body listening, i.e., active listening to the body for insight; (8) trusting, i.e., experiencing one’s body as being safe and trustworthy (Mehling et al., 2012).

This study aimed to address previous inconsistency between ECI and cognitive theories in an emotional process perspective, which requires multiple components to disentangle. Thus, we profiled emotional reactivity and IS using the sub-dimensions to capture as much information as possible. Self-reported questionnaires, including the IS, dispositional emotional reactivity, and depressive symptoms were assessed. Two-stage cluster analysis was used to capture the individual differences in IS. The three dimensions of emotional reactivity (ease of activation, intensity, and duration) were entered as independent variables to predict depressive symptom severity using bootstrapping hierarchical regression, controlling for gender and response bias (i.e., social desirability) separately among each IS cluster.

## 2. Methods

### 2.1. Participants

There were 270 study participants, mean aged 22.10 ± 2.77 years, and 188 of them were females (69.6%). Online advertisements were used to recruit participants. Those aged between 20 and 64 years, whose mother tongue was traditional Chinese, who had normal vision (with or without correction), who were not diagnosed with mental disorders that might influence reality testing or cognitive ability, such as schizophrenia or dementia were eligible for participation.

### 2.2. Measurements

#### 2.2.1. Multidimensional assessment of interoceptive awareness

The MAIA assesses the IS (Mehling et al., 2012). This 32-item self-report questionnaire contains 8 scales, ranging from 3 to 7 items in each scale. Each subscale is described in the IS section in the introduction. Each item describes a response to bodily sensations (e.g., I trust my body sensations), and respondents rate how often they relate to their own body in that way on a six-point Likert scale (0 = never, 5 = always). A higher mean score on each subscale means higher IS tendency in the given domain. The internal consistency of each scale ranged from 0.66 to 0.82 in the original version (Mehling et al., 2012) and from 0.46 to 0.88 in the Chinese version (Lin et al., 2017). The MAIA also exhibits acceptable construct validity among different samples and is thus recommended for assessing IS (Mehling et al., 2012; Lin et al., 2017; Pollatos and Herbert, 2018).

#### 2.2.2. Perth emotional reactivity scale - short form

The PERS short form (PERS-S) is a self-reported questionnaire that assesses emotional reactivity at the trait level (Preece et al., 2018). It contains two scales: general positive reactivity (PERS-GPR) and negative reactivity (PERS-GNR). Each scale can be further divided into ease of activation (“Act,” e.g., I tend to get happy very easily), intensity (“Int,” e.g., I experience positive mood very strongly), and duration (“Dur,” e.g., I can remain enthusiastic for quite a while) of emotional reactions, resulting in a six-factor structure. It uses a five-point Likert scale (1 = very unlike me, 5 = very like me). A higher score indicates stronger emotional reactivity. The original version contains 30 items, and there are 18 short-form items. The original version exhibited good internal reliability, factor structure, and concurrent validity (Becerra et al., 2017). The PERS-S showed a consistent factor structure to PERS, good concurrent validity, and the Cronbach’s alpha of PERS-GPR and PERS-GNR were 0.92 and 0.91, respectively (Preece et al., 2018). Overall, the PERS-S showed good psychometric characteristics.

#### 2.2.3. Beck depression inventory-II

The beck depression inventory-II (BDI-II) contains 21 symptoms of depression, and respondents have to choose the most befitting ones based on their experiences over the previous 2 weeks. Each item score ranges from 0 to 3, with a higher total score indicating more severe depressive symptoms. Individuals with a total score of 0–13 were classified as minimal depression, 14–19 as mild, 20–28 as moderate, and 29–63 as severe (Beck et al., 1996). This widely used assessment has been translated into various languages, and psychometric data have been established for different cultures (Wang and Gorenstein, 2013). The Chinese version of the BDI-II also exhibited good reliability and validity (Wu and Huang, 2014).

#### 2.2.4. Marlowe-Crowne social desirability scale - short form

This scale is a shorter version of the widely used Marlowe-Crowne Social Desirability Scale. This 13-item forced choice scale assesses social desirability (Reynolds, 1982). The responses are scored as 0 or 1 for each item (e.g., I sometimes feel resentful when I do not get my way), and a higher score indicates a higher tendency to bias responses for social approval. The short form exhibited good psychometric properties and was highly correlated with the original form (Reynolds, 1982; Barger, 2002).

### 2.3. Procedure

The data are part of the general assessment of the Human Project from Mind, Brain, and Learning (HPMBL) in Taiwan. The HPMBL study was approved by the Research Ethics Committee of Chengchi University (NCCU-REC- 201810-I074). HPMBL is a database project, which began in 2018, about the psychological phenomena of adults in modern life, and more than 60 constructs were collected using computerized behavioral tests and self-reported questionnaires. In order to avoid possible confounding, there were six versions of questionnaire sequences with a pseudo-random presentation. Participants provided informed consent for the entire procedure before the first round of data collection and subsequently scheduled the next round. Participants received 400–600 NTD (approximately 13–20 USD) for each data collection process according to the amount of data collected. The payments were different because some data collection processes were longer than the others.

### 2.4. Statistical analysis

A partial correlation analysis (controlled for gender and social desirability) was conducted. Two-stage clustering analysis was used to cluster IS tendency (i.e., scores of the eight subscales of MAIA). Agglomerative hierarchical clustering (Ward’s method) was used in Stage 1 and K-means (*K* = 3) in Stage 2. Chi-square, ANOVA, and Bonferroni *post hoc* comparisons were used to examine the differences in gender and social desirability scores among the IS groups. ANCOVA (controlled for gender and social desirability) and *post hoc* contrasts were used to examine the differences in PERS-S and BDI-II scores between the IS groups. Bootstrapping regression (2000 sampling) of PERS-S subscales on the BDI-II, controlling for gender and social desirability, was conducted among the three IS groups. Subscales for negative and positive reactivity were entered into separate models. All statistical analyses were conducted using IBM SPSS Statistics software. Because there were multiple analyses in this study, the alpha was set as 0.01 to prevent elevated type I error, thus, a BCa 99% confidence interval (CI) was used in Bootstrapping regression.

## 3. Results

### 3.1. Partial correlation between all measures

Table 1 presents the partial correlations (controlled for sex and social desirability). Except for Not-Distracting and Not-Worrying, all MAIA subscales were correlated to each other as well as with positive reactivity. Depressive symptoms were significantly negatively correlated to NAIA subscales of Not-Worrying, Self-Regulation, Trusting and all PERS-S positive subscales, and positively correlated to all PERS-S negative subscales.

**Table 1:** Partial correlation between all measures.

	2	3	4	5	6	7	8	9	10	11	12	13	14	15	16	17
1. MAIA-Noticing	0.04	−0.10	**0.40**	**0.51**	**0.28**	**0.38**	**0.29**	**0.28**	**0.23**	**0.27**	**0.25**	0.05	0.03	0.09	0.02	−0.01
2. MAIA-Not-Distracting		0.06	−0.01	0.00	−0.10	−0.05	−0.02	−0.06	−0.09	−0.05	−0.02	−0.06	−0.09	−0.02	−0.04	0.02
3. MAIA-Not-Worrying			**0.20**	−0.13	0.15	−0.09	−0.03	0.02	0.08	−0.05	0.02	**−0.31**	**−0.22**	**−0.30**	**−0.31**	**−0.19**
4. MAIA-Attention Regulation				**0.60**	**0.63**	**0.55**	**0.48**	**0.31**	**0.23**	**0.33**	**0.28**	**−0.20**	**−0.19**	−0.12	**−0.23**	−0.13
5. MAIA-Emotional Awareness					**0.47**	**0.63**	**0.38**	**0.29**	**0.19**	**0.34**	**0.26**	0.04	0.01	0.11	−0.02	0.03
6. MAIA-Self-Regulation						**0.66**	**0.51**	**0.30**	**0.21**	**0.29**	**0.30**	**−0.26**	**−0.24**	**−0.22**	**−0.26**	**−0.20**
7. MAIA-Body Listening							**0.52**	**0.27**	**0.19**	**0.29**	**0.25**	−0.07	−0.11	0.00	−0.09	−0.04
8. MAIA-Trusting								**0.50**	**0.45**	**0.45**	**0.44**	**−0.29**	**−0.28**	**−0.24**	**−0.26**	**−0.38**
9. PERS-GPR									**0.89**	**0.90**	**0.91**	**−0.22**	**−0.26**	−0.16	−0.16	**−0.37**
10. PERS-PAct										**0.71**	**0.72**	**−0.22**	**−0.22**	**−0.17**	**−0.18**	**−0.38**
11. PERS-PInt											**0.73**	−0.13	**−0.19**	−0.06	−0.09	**−0.27**
12. PERS-PDur												**−0.24**	**−0.27**	**−0.19**	**−0.16**	**−0.36**
13. PERS-GNR													**0.89**	**0.92**	**0.85**	**0.56**
14. PERS-NAct														**0.73**	**0.63**	**0.53**
15. PERS-NInt															**0.68**	**0.51**
16. PERS-NDur																**0.44**
17. BDI																

The bold *r* values were significant: absolute *r* > 0.20, *p* < 0.001; absolute *r* > 0.16, *p* < 0.01.

### 3.2. IS clustering and description of each group

According to the results of hierarchical clustering in Stage 1, we tested K as 2 and 3 in Stage 2. With K = 2. The “Not-Worrying” and “Not-Distracting” subscales were not significantly different between groups. With *K* = 3, 7 of the 8 subscales of MAIA were significantly different (*p*s < 0.001) between groups, and the “Not-Distracting” subscale was not significantly different (*p* = 0.067). Thus, *K* = 3 was used to capture various groups. Table 2 lists the final cluster centroids. According to the centroids, the three groups were as follows: the “high IS” group (*N* = 51), which was characterized by a high awareness of bodily sensations and emotions as well as the ability to self-regulate using those sensations; the “low IS” group (*N* = 101), the members of which did not orient themselves to their bodies and spent little effort on self-regulation; the “worriers” (*N* = 118), who showed high awareness of bodily sensations but tended to worry about those sensations and failed to self-regulate.

**Table 2:** Final centroids of three MAIA clusters.

	High IS *N* = 51	Low IS *N* = 101	Worrier *N* = 118	*F*	*p*
Noticing	4.01	3.16	3.62	34.27	<0.001
Not-distracting	2.18	2.03	1.90	2.74	0.067
Not-worrying	2.01	1.67	1.36	13.42	<0.001
Attention regulation	3.70	2.18	2.91	130.47	<0.001
Emotional awareness	4.24	2.67	3.62	126.44	<0.001
Self-regulation	3.81	1.92	3.00	161.71	<0.001
Body listening	4.12	1.81	3.23	246.42	<0.001
Trusting	4.17	2.64	3.84	88.88	<0.001

Table 3 presents the group differences in the study variables. Social desirability and most emotional reactivity subscales were significantly different among the three IS clusters.

**Table 3:** Differences between three IS clusters.

	High IS group (H)	Low IS group (L)	Worrier (W)			
	*N* (%)	*N* (%)	*N* (%)	*χ* ^2^	*p*	
**Gender**				0.92	0.631	
Female	33 (64.7)	73 (72.3)	82 (69.5)			
Male	18 (35.3)	28 (27.7)	36 (30.5)			
**BDI-categories**						
Minimal	43	77	92	5.87	0.438	
Mild	2	8	13			
Moderate	4	8	8			
Severe	1	6	2			
	**M ± SD**	***M*** ** ± ** ***SD***	***M*** ** ± ** ***SD***	***F***	***p***	**Post hoc** ^a^
SDS	6.42 ± 2.81	4.05 ± 2.54	4.97 ± 2.23	15.59	<0.001	H > W > L
	**M ± SD**	***M*** ** ± ** ***SD***	***M*** ** ± ** ***SD***	***F*** ^b^	***p***	**Contrasts**
PERS-GPR	36.56 ± 6.45	29.51 ± 6.59	33.02 ± 5.42	19.63	<0.001	H > W > L
PERS-PAct	12.44 ± 2.15	10.55 ± 2.37	11.56 ± 2.05	10.17	<0.001	H > W > L
PERS-PInt	12.32 ± 1.82	9.74 ± 2.47	11.03 ± 1.88	22.98	<0.001	H > W > L
PERS-PDur	11.80 ± 2.44	9.22 ± 2.39	10.42 ± 2.23	15.95	<0.001	H > W > L
PERS-GNR	23.28 ± 8.69	29.11 ± 7.03	28.16 ± 6.73	5.99	0.003	H < W ≈ L
PERS-NAct	7.90 ± 3.50	10.20 ± 2.71	9.71 ± 2.58	5.93	0.003	H < W ≈ L
PERS-NInt	8.06 ± 3.55	9.368 ± 2.85	9.47 ± 2.78	2.29	0.103	
PERS-NDur	7.32 ± 2.55	9.23 ± 2.31	8.98 ± 2.28	7.33	0.001	H < W ≈ L
BDI	6.92 ± 8.11	10.57 ± 8.97	8.76 ± 6.99	1.21	0.300	

^a^Bonferroni test; ^b^ANCOVA controlling for gender and SDS.

### 3.3. Regression of emotional reactivity on depressive symptoms of three IS groups

#### 3.3.1. Positive emotional reactivity and depression

The coefficients of bootstrapped regression are presented in Table 4. The predictive patterns of three IS groups were differentiable. The positive reactivity characteristics did not significantly predict depressive severity among high IS cluster (*R*^2^ = 0.22, Adj *R*^2^ = 0.13, *F*(5, 43) = 2.34, *p* = 0.054). Among the low IS cluster, the lower score of the PAct marginally significantly predicted more severe depressive symptoms (*B* = −1.51, *p* = 0.015, BCa 99% CI [−3.02, −0.18]). Among Worriers, a lower PDur score significantly predicted more severe depressive symptoms (*B* = −0.93, *p* = 0.008, BCa 99% CI [−1.84, 0.09]). In addition, the effect of full regression after controlling for SDS and gender among Worriers cluster was only marginally significant (Δ*R*^2^ = 0.09, Δ*F*(3, 109) = 3.71, *p* of Δ*F* = 0.014). Emotional intensity did not predict depressive symptoms among all IS clusters.

**Table 4:** Bootstrap coefficients of positive and negative emotional reactivity on depression.

	High IS (*N* = 51)						Low IS (*N* = 101)						Worrier (*N* = 118)					
	B	Bias	SE	*p*	BCa 99% CI		B	Bias	SE	*p*	BCa 99% CI		B	Bias	SE	*p*	BCa 99% CI	
					Lower	Upper					Lower	Upper					Lower	Upper
**Positive emotional reactivity**																		
*Model 1*																		
Constant	10.15	−0.003	2.52	<0.001	4.28	17.37	14.21	−0.06	1.92	<0.001	9.29	19.10	11.26	−0.05	1.54	<0.001	7.44	15.29
Gender	−1.58	0.06	2.57	0.547	−6.97	6.58	−0.66	0.08	2.06	0.767	−5.34	5.35	−0.74	0.05	1.32	0.586	−3.83	2.80
SDS	−0.42	−0.002	0.34	0.217	−1.38	0.48	−0.840	0.01	0.44	0.056	−2.00	0.42	−0.46	0.01	0.26	0.081	−1.24	0.21
	*R* ^2^ = 0.03, Adj *R*^2^ = −0.01, *F*(2, 46) = 0.77, *p* = 0.471						*R* ^2^ = 0.05, Adj *R*^2^ = 0.03, *F*(2, 92) = 2.63, *p* = 0.078						*R* ^2^ = 0.02, Adj *R*^2^ = 0.01, *F*(2, 112) = 1.29, *p* = 0.280					
*Model 2*																		
Constant	19.91	0.58	9.41	0.047	−1.86	47.00	36.78	0.15	5.02	<0.001	24.10	51.30	20.64	−0.15	4.60	<0.001	8.52	32.60
Gender	−2.28	−0.35	2.19	0.318	−7.40	2.51	−3.87	0.03	1.96	0.046	−8.55	1.39	−1.64	0.10	1.51	0.280	−5.27	2.57
SDS	−0.21	−0.04	0.41	0.624	−1.21	0.77	−0.85	0.01	0.35	0.020	−1.85	0.15	−0.23	−0.004	0.27	0.402	−1.01	0.41
PAct	−1.21	−0.05	1.06	0.256	−4.50	1.02	**−1.51**	**−0.02**	**0.57**	**0.015**	**−3.02**	**−0.18**	−0.40	0.01	0.47	0.384	−1.64	0.90
PInt	1.38	−0.20	1.03	0.230	−0.97	3.48	−0.19	0.03	0.45	0.668	−1.26	1.32	0.37	−0.002	0.45	0.415	−0.72	1.53
PDur	−1.09	0.24	1.14	0.391	−4.13	2.55	−0.43	−0.03	0.53	0.412	−1.88	0.82	**−0.93**	**0.01**	**0.34**	**0.008**	**−1.84**	**0.09**
	*R* ^2^ = 0.22, Adj *R*^2^ = 0.13, *F*(5, 43) = 2.34, *p* of *F* = 0.054 Δ*R*^2^ = 0.19, Δ*F*(3, 43) = 3.34, *p* of Δ*F* = 0.027						*R* ^2^ = 0.30, Adj *R*^2^ = 0.26, *F*(5, 89) = 7.54, *p* of *F* < 0.001 Δ*R*^2^ = 0.24, Δ*F*(3, 89) = 10.29, *p* of Δ*F* < 0.001						*R* ^2^ = 0.11, Adj *R*^2^ = 0.07, *F*(5, 109) = 2.78, *p* of *F* = 0.021 Δ*R*^2^ = 0.09, Δ*F*(3, 109) = 3.71, *p* of Δ*F* = 0.014					
**Negative emotional reactivity**																		
*Model 1*																		
Constant	10.15	0.08	2.49	<0.001	3.56	17.25	14.39	−0.03	1.90	<0.001	9.32	19.18	11.25	−0.04	1.55	<0.001	7.56	15.33
Gender	−1.58	0.13	2.61	0.561	−7.17	6.66	−1.02	0.05	2.05	0.623	−5.87	4.84	−0.74	0.00	1.31	0.575	−3.90	2.63
SDS	−0.42	−0.01	0.33	0.199	−1.30	0.42	−0.87	0.001	0.44	0.044	−1.94	0.34	−0.46	0.01	0.26	0.077	−1.22	0.24
	*R* ^2^ = 0.03, Adj *R*^2^ = −0.01, *F*(2, 46) = 0.77, *p* = 0.471						*R* ^2^ = 0.06, Adj *R*^2^ = 0.04, *F*(2, 95) = 3.02, *p* = 0.053						*R* ^2^ = 0.02, Adj *R*^2^ = 0.01, *F*(2, 112) = 1.23, *p* = 0.280					
*Model 2*																		
Constant	−6.10	−0.10	3.97	0.161	−16.51	3.70	−10.62	0.19	5.95	0.087	−26.00	4.90	−4.80	−0.19	3.13	0.129	−13.32	2.69
Gender	−1.23	−0.09	1.96	0.540	−6.00	3.32	1.09	0.02	1.70	0.535	−2.97	5.71	−0.57	0.02	1.21	0.641	−3.52	2.66
SDS	0.20	−0.001	0.32	0.538	−0.53	1.00	−0.08	−0.03	0.43	0.869	−1.05	1.04	−0.42	0.02	0.26	0.105	−1.21	0.26
NAct	**1.53**	**−0.12**	**0.53**	**0.009**	**0.34**	**2.53**	0.88	−0.02	0.37	0.022	−0.04	1.82	0.59	0.01	0.33	0.079	−0.29	1.43
NInt	0.60	0.07	0.46	0.178	−0.45	2.27	0.85	0.004	0.44	0.060	−0.25	2.14	0.23	−0.01	0.30	0.429	−0.59	0.92
NDur	−0.65	0.06	0.67	0.374	−2.63	1.10	0.45	0.01	0.50	0.374	−0.96	1.81	**0.89**	**0.02**	**0.36**	**0.014**	**−0.02**	**1.97**
	*R* ^2^ = 0.55, Adj *R*^2^ = 0.50, *F*(5, 43) = 10.65, *p* of *F* < 0.001 Δ*R*^2^ = 0.52, Δ*F*(3, 43) = 16.72, *p* of Δ*F* < 0.001						*R* ^2^ = 0.35, Adj *R*^2^ = 0.31, *F*(5, 92) = 9.69, *p* of *F* < 0.001 Δ*R*^2^ = 0.29, Δ*F*(3, 92) = 13.35, *p* of Δ*F* < 0.001						*R* ^2^ = 0.30, Adj *R*^2^ = 0.27, *F*(5, 109) = 9.48, *p* of *F* < 0.001 Δ*R*^2^ = 0.28, Δ*F*(3, 109) =14.63, *p* of Δ*F* < 0.001					

The bold coefficients were significant or marginally significant according to *p* value and/or BCa 99% CI.

#### 3.3.2. Negative emotional reactivity and depression

The coefficients of bootstrapped regression are presented in Table 4. The predictive patterns of three IS groups were differentiable. Higher NAct score predicted more severe depressive symptoms among the high IS cluster (*B* = 1.53, *p* = 0.009, BCa 99% CI [0.34, 2.53]). None of negative reactivity subscales significantly predict depressive symptoms among low IS cluster, though the full regression was significant (*R*^2^ = 0.35, Adj *R*^2^ = 0.31, *F*(5, 92) = 9.69, *p* of *F* < 0.001). Among Worriers, higher NDur score tend to predict higher depressive symptoms (*B* = 0.89, *p* = 0.014, BCa 99% CI [−0.02, 1.97]). Emotional intensity did not predict depressive symptoms among all IS clusters.

## 4. Discussion

This study provides deeper insights into subjective emotional reactivity and the important role of IS in depression. Using cluster analysis, the participants were divided into three groups according to their IS characteristics. The predictive patterns of emotional reactivity on depressive symptoms were differentiable between three clusters. Too easy to be activated negatively among high IS individuals is elevating the risk to feel depressed. In contrast, too hard to be activated positively among low IS individuals is problematic. To feel positive shorter and to feel negative longer is at risk among worriers. Though the positive insufficiency and negative exaggerate effect suggested by cognitive theories (Beck, 1979; Gross and Jazaieri, 2014) were primarily supported in our study, however, not among every IS cluster. Positive insufficiency was supported among the low IS and worriers clusters; in contrast, negative exaggeration was supported among the high IS and worriers clusters. The results implied a moderating role of IS in depression psychopathology.

Results indicated depression is associated with emotional reactivity, also related to three subscales of MAIA. Depressive symptoms were significantly associated with all dimensions of positive reactivity (negative relationship) and negative reactivity (positive relationship) (Table 1). This is consistent to cognitive theories (Beck, 1979; Gross and Jazaieri, 2014). Depressive symptoms were also related to Not-Worrying, Self-Regulation, and Trusting subscales of MAIA (Table 1). It is consistent to the pathological importance of repetive negative thinking (Treynor et al., 2003) and emotion regulation (Hofmann et al., 2012; Gross and Jazaieri, 2014) in depression, as well as the therapeutic efficacy of mindfulness in which trusting bodily experiences is an important emphasis (Shapiro et al., 2006). Neither the depressive symptom nor the depressive categories were associated with IS clusters (Table 3). This finding supported that IS plays a moderating rather than direct role in emotion-related psychopathology of depression.

IS associated with the predictive patterns of positive reactivity on depression. Positive insufficiency had no effect on depression in the high IS group. This may have resulted from the ceiling effect wherein positive reactivity was significantly higher in the high IS group (Table 3). In contrast, difficulty in activating positive feelings in the low IS group was marginally associated with higher depression. With less attention and usage of bodily sensations, individuals may react less to positive things. This is consistent with the finding that inaccurate perception of heartbeats is associated with positive insufficiency in depression (Dunn et al., 2010; Furman et al., 2013). Insufficient persistence of positive feelings contributed to depressive severity among worriers, suggesting that with negative processing of bodily sensations, their difficulty in sustaining positive feelings contributes to depression. It is also possible that with a general tendency to worry, the worriers suffered from less persistent positive feelings due to the general negative cognitive process, as suggested by cognitive theories of depression (Beck, 1979).

Negative reactivity may affect depression differently according to individual differences in IS. In the high IS group, ease of activation of negative emotions was linked to higher depression. With a strong tendency to process bodily sensations and being emotionally aware of it, too easy to be negatively activated may lead high IS individuals to process negative emotional feelings when encounter every new bad situation. Depressive problem may be accumulated by those feelings. In contrast, among the worriers, longer duration tended to be associated with higher depression. It is possible that because worriers tend to interpret their bodily sensations negatively, they are able to use those sensations to regulate emotions lesser. Thus, their negative feelings persist and continuously contribute to depression. Consistently, while Not-Worrying is negatively associated with emotional awareness, it is positively related to self-regulation (Table 1). This result implies that individuals tend to worry about their bodily sensations; they may be aware of their negative emotions, but fail to regulate emotions through this awareness. This process is similar to brooding in depressive rumination (Treynor et al., 2003). Additionally, this is consistent with the finding that the “Not-Worrying” subscale is associated with the greatest difficulty in emotion regulation sub-dimensions compared to the other seven subscales of the MAIA (Mehling et al., 2012).

Our findings also raise the possibility that psychological connection to bodily sensation may have a stronger association with positive emotions than with negative emotions. Six of the eight subscales of the MAIA (except for Not-Distracting and Not-Worrying) were correlated with positive reactivity; however, only three of the eight subscales were related to negative reactivity (Table 1). This implies that the processing of positive and negative emotions may not be completely parallel in humans. From an evolutionary perspective, feelings are built-in in emotions (James, 1922; Levenson, 1994). However, we may have been “trained” extensively in thinking negatively as a human with complex cognitive functions. People worry because we want to avoid future errors or ruminate to try to compensate for past mistakes (Freeston et al., 1994; Papageorgiou and Wells, 2003; Nolen-Hoeksema et al., 2008). In contrast, we are less likely to “practice” thinking positively on purpose. Hence, positive emotions may retain more evolutionary attributes. Studies of positive emotions would open more gates to understanding the nature of emotions.

The results suggest an individualized intervention plan for depression that considers IS characteristics. For those with high IS, decreasing negative arousal could be incorporated, such as relaxation training. For those with low IS, behavioral activation may be helpful in activation positive emotions. For individuals who tend to worry about their bodily sensations, components related to sustaining negative emotions or extending positive emotions, such as cognitive therapy, may be helpful. In addition, interventions that increase IS may be used to address positive insufficiency since it was not a problem among high IS cluster. Mindfulness and biofeedback may be candidates for increasing IS because of their purpose of enhancing mind–body connections (Shapiro et al., 2006; Frank et al., 2010; Peper et al., 2010; Sze et al., 2010). Evidence has also shown a correlation between mindfulness and IS (Hanley et al., 2017) and IS enhancement due to mindfulness training (Fissler et al., 2016).

Though it was not our main purpose, the MAIA profiling of three clusters may have some suggestion to the conceptualization of IS. Recently, Ferentzi et al. (2021) have found a strong general factor of MAIA (MAIA-g) across MAIA subscales except for Not-Worrying and Not-Distracting based on factor analysis and criterion validity. This is similar to our results that Not-Distracting was not effectively contributed in cluster analysis, and the Not-Worrying was distinct in classifying the worriers cluster while other six subscales (i.e., MAIA-g) were mainly contributed in differentiating high and low IS (Table 2). Drawing from our findings of a distinct predictive pattern of worriers, it is implied that Not-Worrying has a non-negligible role in IS conceptualization, but may not be in the same place with other six subscales. Additionally, Not-Distracting may need further examination in the IS conceptualization, as it was cautioned by the Ferentzi et al. (2021).

Some limitations of this study should be addressed by the future studies. Owing to the community sample, a majority of participants in this study did not disturbed by depressive problem (Table 3). Thus, our results cannot be applied to patients with major depressive disorder. Studies incorporating more severely depressed participants may capture the whole picture of IS and depression. For example, a review by Dunn et al. (2007) suggested an inverted U relationship between “how accurate one’s judgement of their own heartbeats is” and depression. Nonlinear correlation may also exist in IS. Although cluster analysis can capture the IS characteristics in a gist of phenomena, it lacks theoretical basis. With complex domains, IS should be considered as a dynamic process rather than a static attribute. A systematic exploration of IS for a structure based on theoretical suggestions will define the “IS characteristics” better, enabling further understanding of its role in the psychopathology of depression. This study only incorporated the subjective domains of emotional reactivity and interoception. Other aspects were missing. The interaction between physiological and psychological domains is important for both constructs. For example, the effect of an individual’s attention and manipulation of bodily sensations is highly affected by the accuracy of the initial perception. Hence, a comprehensive study examining these constructs should be conducted.

## Data availability statement

Raw data were generated at the Research Center for Mind, Brain and Learning, Chengchi University. Derived data supporting the findings of this study are available from the corresponding author NSY on request.

## Ethics statement

The studies involving human participants were reviewed and approved by Research Ethics Committee of National Chengchi University. The patients/participants provided their written informed consent to participate in this study.

## Author contributions

Y-HH and Y-TH collected the data. Y-HH analysed the data and drafted the article. N-SY acquired the funding. All authors contributed to the study concept and design, edited the manuscript and approved its final version for submission.

## Funding

This work was supported by “The Human Project from Mind, Brain and Learning” of NCCU from the Higher Education Sprout Project by the Ministry of Education in Taiwan.

## Conflict of interest

The authors declare that the research was conducted in the absence of any commercial or financial relationships that could be construed as a potential conflict of interest.

## Publisher’s note

All claims expressed in this article are solely those of the authors and do not necessarily represent those of their affiliated organizations, or those of the publisher, the editors and the reviewers. Any product that may be evaluated in this article, or claim that may be made by its manufacturer, is not guaranteed or endorsed by the publisher.
